# “Musical dish” efficiently induces osteogenic differentiation of mesenchymal stem cells through music derived microstretch with variable frequency

**DOI:** 10.1002/btm2.10291

**Published:** 2022-01-25

**Authors:** Qiulin He, Junxin Lin, Fanghao Zhou, Dandan Cai, Yiyang Yan, Yejie Shan, Shufang Zhang, Tiefeng Li, Xudong Yao, Hongwei Ouyang

**Affiliations:** ^1^ Dr. Li Dak Sum & Yip Yio Chin Center for Stem Cells and Regenerative Medicine, and Department of Orthopedic Surgery of the Second Affiliated Hospital Zhejiang University School of Medicine Hangzhou China; ^2^ Zhejiang University‐University of Edinburgh Institute Zhejiang University School of Medicine, and Key Laboratory of Tissue Engineering and Regenerative Medicine of Zhejiang Province Zhejiang University School of Medicine Hangzhou China; ^3^ Center for X‐Mechanics, Department of Engineering Mechanics Zhejiang University Hangzhou China; ^4^ China Orthopedic Regenerative Medicine Group (CORMed) Hangzhou China; ^5^ The Fourth Affiliated Hospital, Zhejiang University School of Medicine Yiwu China; ^6^ Department of Sports Medicine Zhejiang University School of Medicine Hangzhou China

**Keywords:** regenerative medicine, stem cell therapies, tissue engineering

## Abstract

Nonuniform microstretching (NUMS) naturally occurs in real bone tissues in vivo, but its profound effects have not been identified yet. In order to explore the biological effects of NUMS and static stretch (uniform stretch [US]) on cells, a new “musical dish” device was developed. Musical signal was used to provide NUMS to cells. More stress fibers, arranging along the long axis of cells, were formed throughout the cells under NUMS, compared with US and untreated control group, although cell morphology did not show any alteration. Whole transcriptome sequencing revealed enhanced osteogenic differentiation of cells after NUMS treatment. Cells in the NUMS group showed a higher expression of bone‐related genes, while genes related to stemness and other lineages were down‐regulated. Our results give insights into the biological effects of NUMS and US on stem cell osteogenic differentiation, suggesting beneficial effects of micromechanical stimulus for osteogenesis. The newly developed device provides a basis for the development of NUMS derived rehabilitation technology to promote bone healing.

## 1 INTRODUCTION

Stretch, as a form of mechanical force, has been widely explored as its advanced functions in not only regulation of cell behaviors^1^, ^2^ but also injury prevention and tissue repair.^3^Various studies have explored the effects of stretch stimulation on cell differentiation by tuning different parameters such as frequency, magnitude, direction, and duration. For example, cyclic stretching with increased amplitude (2.0%, 3.5%, and 5.0%) and constant frequency (0.5 Hz) was exploited to promote osteogenic differentiation of cells.^4^ Uniaxial stretching with different amplitude (8% and 12%) were performed on cells, which demonstrated that higher amplitude is required for superior expression of tenogenic genes.^5^ Stretch has also been used for promoting tissue regeneration. For example, cyclic stretch (15%, 1 Hz) delivered in TGF‐β1‐loaded collagen scaffold enhanced tendon regeneration in both mechanical properties and organizational structure.^6^ Static stretch (33%) was identified as a necessary factor for hair regeneration by regulating M2 phenotype of macrophages and activating stem cells.^7^ Although many studies have been performed to reveal the effects of stretch, most of the research focused on stretch with a high magnitude (around 5%–15%), which is far from those forces observed the in vivo environment.

Stretch is nonuniform with variable frequency and magnitude in various tissues such as lung,^8^ heart,^9^ brain,^10^ and skeletal system. Among them, stretch in bone tissues has drawn the most attention for clinical application, as bone tissues are continuously exposed to stretch stimulation during physiological activities. The structure and function of bone tissues relies on proper amount of stretch stimulation, while lack of exercises would result in the reduction of bone volume and osteoporosis.^11^ In clinical application, distraction osteogenesis (DO), a surgery procedure involving cutting and separating bone and providing stretch at both ends, promotes bone repair and regeneration through stretch. For the stretch in DO, the frequency is very low (about four times per day) and the magnitude is large (over 1 mm).^12^, ^13^ In contrast, under physiological condition, bone tissues are exposed to micro‐stretch with variable frequency.^6^, ^14^ However, the biological effects of such microstretch have not been identified yet.

To artificially reproduce the biophysical condition, cell‐stretching devices have been widely developed. Commercial Flexercell Tension devices and two‐layer microfluidic stretching system are normally used to produce static or cyclic stretch with a constant frequency.^4^, ^6^, ^15^, ^16^, ^17^ But these devices, in which stretch is caused by the deformation of the gas channels through vacuum pressure and positive air pressure, is unable to efficiently generate stretch with variable frequencies and low amplification similar to the in vivo bone condition. Advanced responsive materials, including thermal actuation relying on the phase transition of materials such as temperature responsive hydrogels^18^ and liquid crystal elastomers,^19^, ^20^ magnetic actuation controlling the deformation of micropillars on a substrate with an external magnetic field,^21^ and electric actuation based on the electric‐mechanical coupling behavior of dielectric elastomer actuators (DEAs), have also been used to provide stretching stimulation. Among them, cell stretchers based on DEAs exhibit performance advantages, including fast response, large deformation, optical transparency, and robust controllability.^22^

Motivated by these findings, here we propose a new design of a DEA‐based cell stretcher, named as “musical dish,” to provide nonuniform microstretching (NUMS) to cells. A thin layer of single‐wall carbon nanotube (SWCNTs) was acted as the high‐voltage electrode with excellent transparency and compliance for optical cell stretching and monitoring. NUMS was applied to cells by converting a musical signal into electrical energy, followed by converting the signal further into mechanical energy via a flexible DE membrane. The effects of NUMS, US, and unstretched control groups on cell morphology, cytoskeletal organization, and osteogenic differentiation were demonstrated. Whole transcriptome sequencing was performed to identify gene expression changes. Based on these results, the “musical dish” system paves the way for the application of NUMS to direct stem cells toward osteogenic phenotypes, further offers a clinical strategy of biomechanical regulation for bone tissue repair. These findings are useful for the development of NUMS derived rehabilitation technology to promote bone healing.

## 2 RESULTS

### 2.1 Fabrication of the musical dish

**FIGURE 1:**
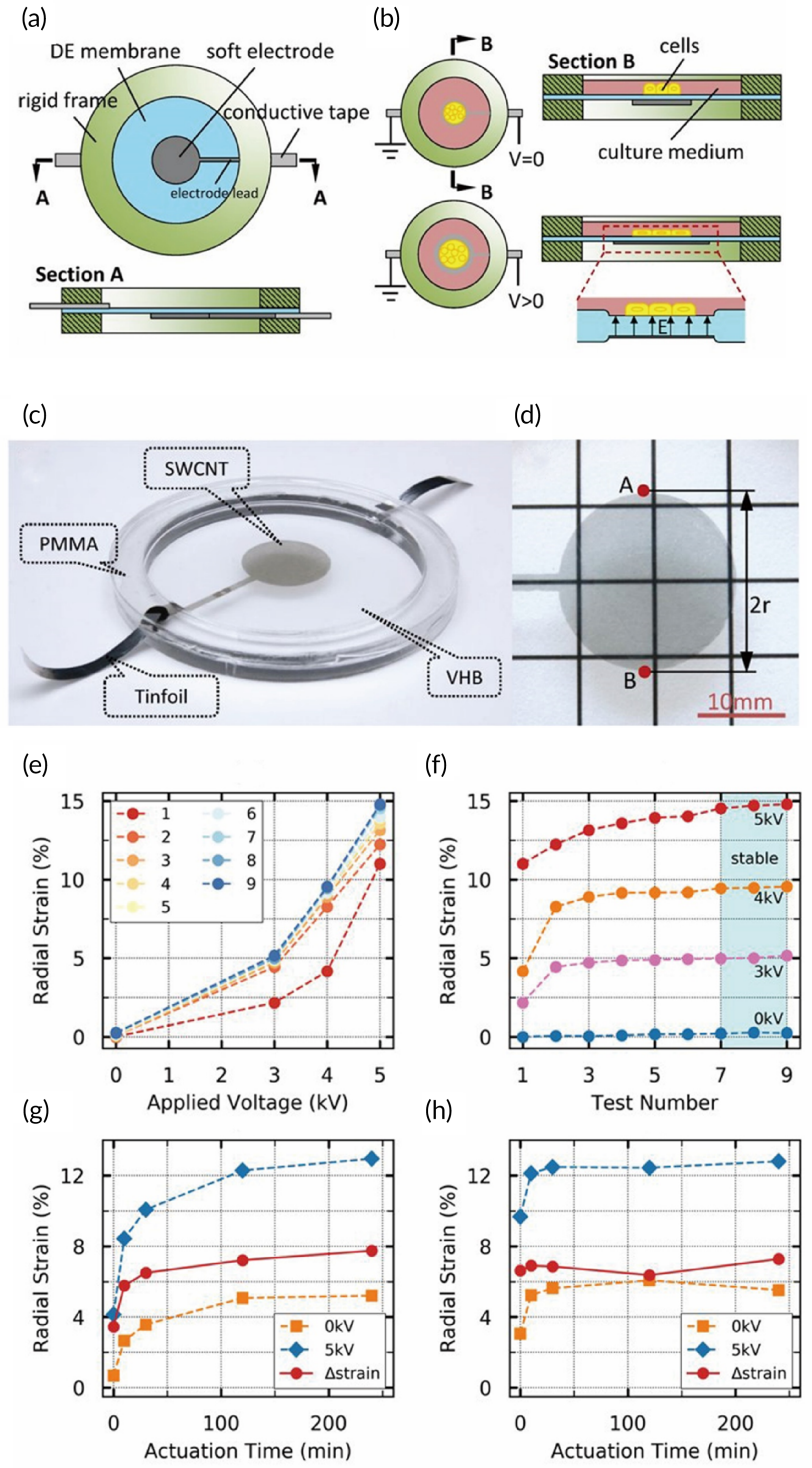
Fabrication and strain characterization of the musical dish. (a) Schematics of the components in the musical dish. (b) The mechanism of cell stretching. (c) Picture of the musical dish. (d) Picture of the transparent electrode above a sheet with square lattice. (e,f) The static radial strain as a function of (e) applied voltage and (f) test number. (g,h) The cyclic radial strain under an alternating voltage between 0 V and 5 kV at (g) 0.15 Hz and (h) 1 Hz. Blue diamond: the average values of the maximum radial strains; orange square: the average values of the minimum radial strains; red circle: the difference between the maximum strains and the minimum strains

The newly developed musical dish was shown in Figure 1a–d. The prestretched DE membrane was coated with a soft electrode (fire wire electrode) in the central area of the bottom and sandwiched by two rigid frames (Figure 1a). The soft electrode was connected to the conductive tape through the electrode lead. The top rigid frame and the DE membrane made up a culture chamber. Culture medium in the chamber acted as ground wire electrode, due to its conductivity (Figure 1b). The cells were seeded in the central circular zone covered by the soft electrode (Figure 1b). DE membrane expanded in plane under the action of electric current, stretching the adhered cells on it (Figure 1b). The transparency of the electrode was shown in Figure 1c,d.

### 2.2 Strain characterization of the musical dish

In the characterizations of the actuating behaviors, the radial strain was calculated through Equation (1). The results were presented as a function of both applied voltage and test number (Figure 1e,f). Due to viscoelasticity, deformations exhibited some difference between different tests. The results obtained from the first test were notably small. However, with the test number increasing, the results finally converged, which revealed that the actuating behavior of the stretcher became stable. Figure 1e,f showed a “warm‐up” of six tests was essential before using the stretcher in static case.

Figure 1g,h presented the relationships between the radial strain and the actuating time at 0.15 Hz and 1 Hz (AC voltage, 0 V–5 kV), respectively. At the beginning, both the maximum and the minimum strain were small. The DEA stretcher with higher actuating frequency reached the stable state earlier. The strain drift arising from viscosity resulted in a steady state of tensile strain ranging from 5% to 13%. This type of strain may be of great interest to study the effects of mechanical stimuli on cell behaviors.

### 2.3 The homogeneity and equiaxiality of the musical dish

**FIGURE 2:**
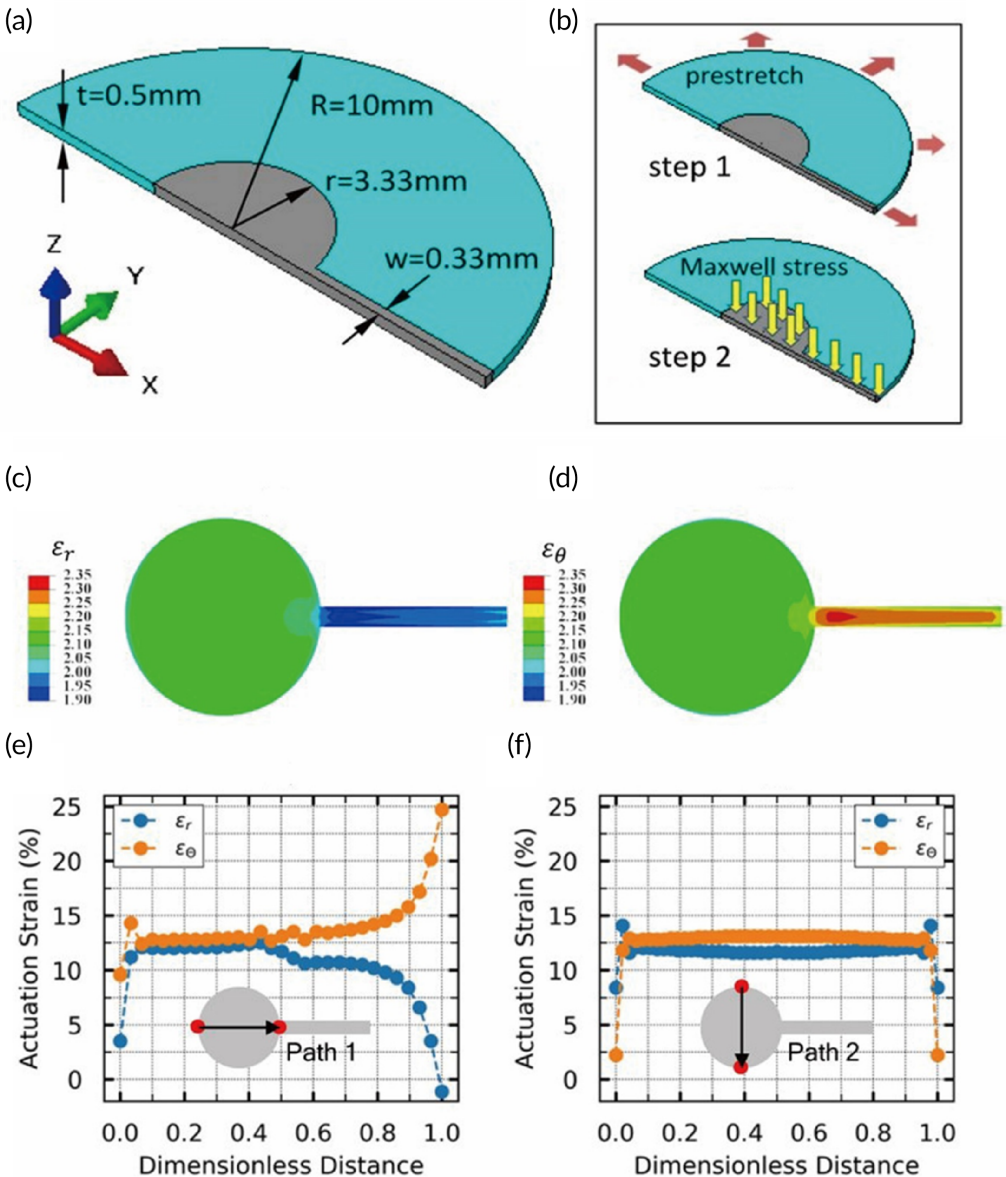
Model analysis of the homogeneity and equiaxiality of the musical dish. (a) Geometry and parameter definition. (b) The prestretch and Maxwell stress loading steps in the finite element analysis (FEA) simulation. (c) The contour of radial strain. (d) The contour of hoop strain. (e) The strain distribution along Path 1. (f) The strain distribution along Path 2

The radial strain and the hoop strain of the active part of the DE membrane were further studied. As only the stretching of the SWCNT electrode lead to the deformation of cells, the model was simplified as a prestretched DE membrane under a local compression induced by an external voltage (Figure 2b). Taking advantage of the symmetry of the geometry, boundary and load, a 1/2 model was established (Figure 2a,b). The strain components contained the contribution of the prestretch in Step 1, so the actuating strain should be obtained by subtracting 2 from the results showed here. As a result, the major area of the central circular zone had an almost uniform and equiaxial distribution of actuating strain (Figure 2c,d). Inhomogeneous deformation was limited only at the small junction between the SWCNT electrode and the electrode lead. The actuating strains along two specific paths were shown in Figure 2e,f. The lowest level of homogeneity and equiaxiality of the actuating strain were measured along Path 1. Due to the coaxiality of the path and the electrode lead, the radial strain in the right part of the path was suppressed to some extent. And there was a steep decrease of radial strain near the end point of the path. The hoop strain exhibited a steep increase instead. Path 2 represented the highly uniform distribution of two strain components. Based on the strain distribution, we concluded that the homogeneity and equiaxiality of the culture zone were overall acceptable.

### 2.4 Establishment of musical dish culture system

**FIGURE 3:**
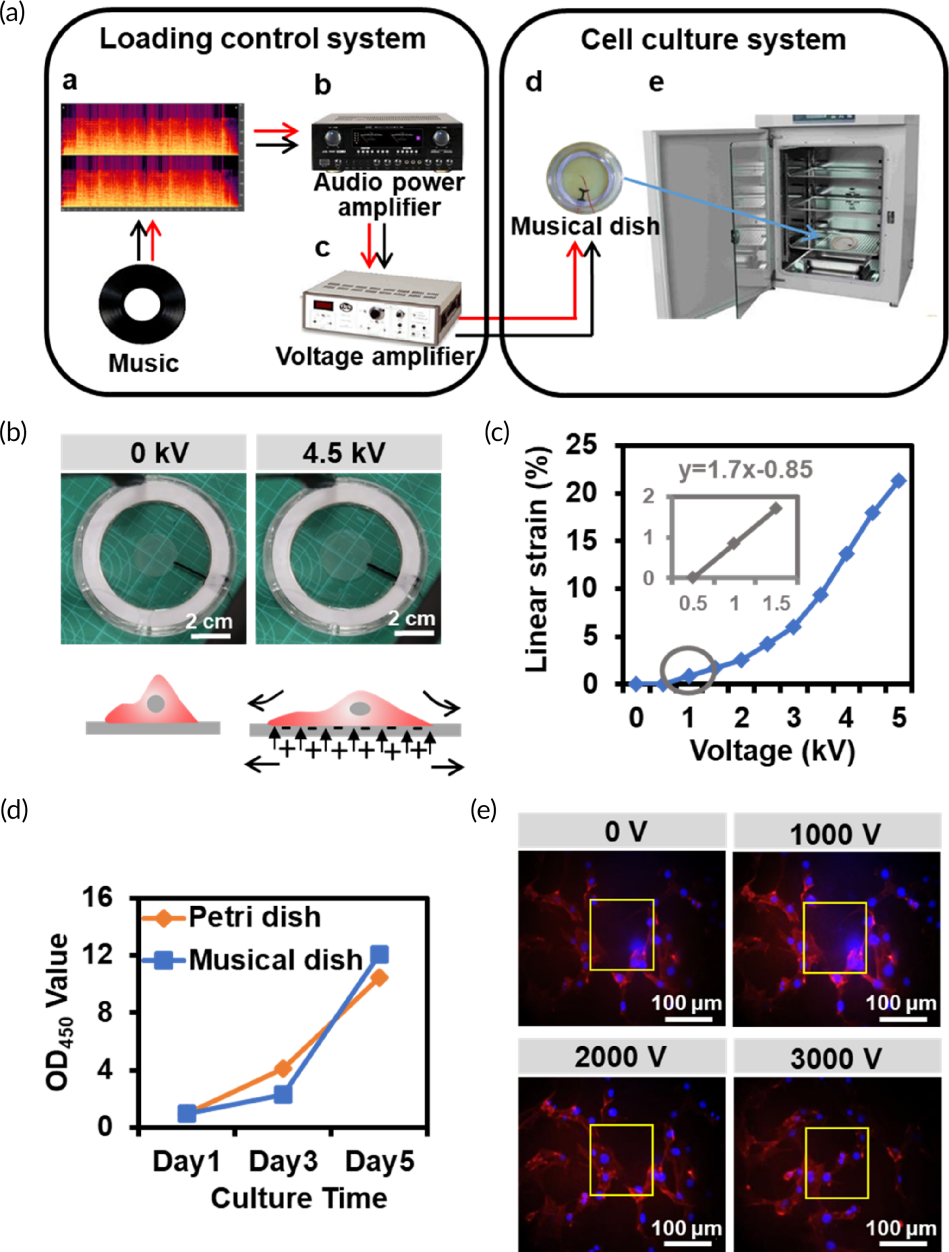
Fabrication and characterization of the musical dish culture system. (a) Diagram of musical dish culture system. (b) Pictures of musical dish at 0 and 4500 V. (c) Linear strain of musical dish from 0 to 5000 V. (d) CCK8 assay for cell proliferation in the musical dish and Petri dish groups between Days 1 and 5. (e) Phalloidin (red) and DAPI (blue) staining to show cell deformation under stretch by musical dish

The musical dish culture system consisted of a loading control system (musical signal, audio power amplifier, and voltage amplifier) and a cell culture system (musical dish) (Figure 3a). The audio power amplifier can recognize and amplify the musical signal and convert them into electric current signal. The electric current signal was transmitted to the voltage amplifier. Then, an electric field was generated in the SWCNT electrode of musical dish by connecting the fire wire electrode to high voltage and the ground wire electrode to low voltage. The SWCNT electrode was deformed under the electric field to enlarge the area and reduce the thickness due to the attraction of heteroelectric charge (Figure 3b).

The linear strain was measured. The results showed that the SWCNT electrode was stretched when applied to the voltage (Figure 3c). Within a certain range (0–3 kV), the linear strain increased slowly with the increase of the voltage, while when the voltage was greater than 3 kV, the linear strain increased significantly. When a voltage of 1 kV was applied, the SWCNT electrode produced a tensile force with the amplitude of less than 1%, which is close to the stretch range in bone tissues.^5^

### 2.5 Cell proliferation and deformation in the musical dish system

To investigate the feasibility of musical dish for cell growth, C3H10T1/2 cell lines were seeded on the musical dish and the Petri dish. During 5 days' observation, cell density distribution did not vary between the two groups (Figure S1). Cell counting kit‐8 (CCK‐8) assay was further used to evaluate the activity of the cells cultured on the musical dish and Petri dish at Days 1, 3, and 5, respectively. The results confirmed that no significant difference in the CCK‐8 assay was observed between the two groups (Figure 3d), suggesting no mechanical cell injury or breakage in the self‐made musical dish.

We then examined whether the deformation of the SWCNT electrode of musical dish can indeed have an impact on cells. Cells seeded on the musical dish were labeled with DAPI (nucleus) and TRITC (cytoskeleton). A voltage of 0, 1, 2, and 3 kV was applied. The results showed that when the system was loaded with a constant voltage, the SWCNT electrode stretched, leading to the dislocation of the nuclear position and the disturbance of the cytoskeleton arrangement (Figure 3e). The movement of cells was increased with the increase of the voltage.

### 2.6 The effect of stretch stimulation on cell morphology and cell cytoskeleton

**FIGURE 4:**
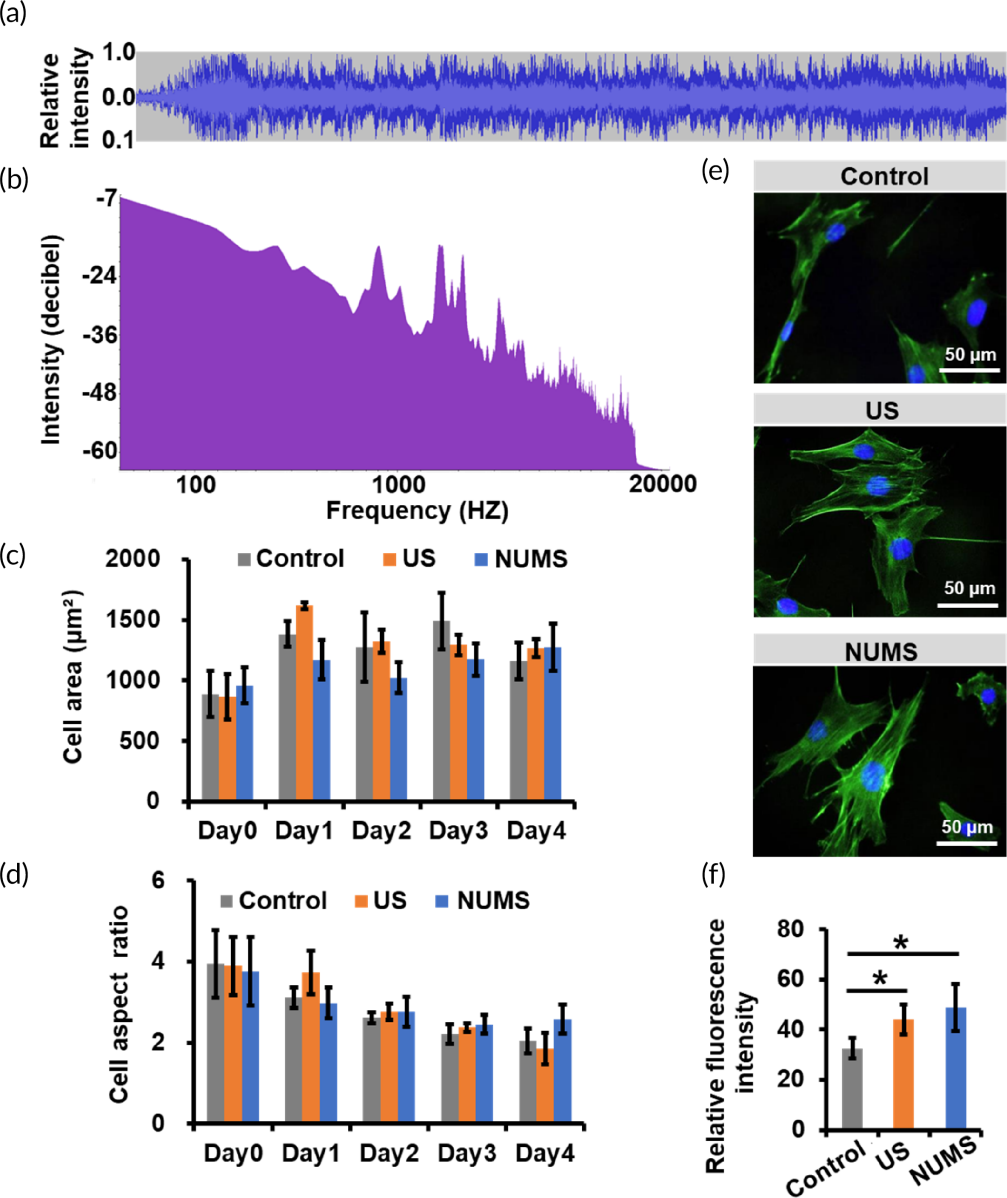
The effect of stretch stimulation on cell morphology and cell cytoskeleton. (a) The relationship between stretching amplitude and time in the NUMS group. (b) The relationship between stretching frequency and intensity of the musical signal. (c,d) Cell area (c) and cell aspect ratio (d) analysis to show cell morphology in the nonuniform microstretching (NUMS), uniform stretch (US), and control group from Day 1 to Day 4 (*N* = 3, *n* > 8). (e,f) Phalloidin/DAPI staining (e) and quantitative analysis (f) to show cell cytoskeleton in the NUMS, US, and control group after stimulated for 6 h (*N* = 3, *n* = 6)

To evaluate the effect of NUMS on cells, 1 kV constant voltage with musical signal was applied to cells (Figure 4a). According to the equation *y =* 1.7x‐0.85 (Figure 3c), a variable stretch was estimated to range from 0.833% to 0.867% (48.73–50.71 μm) along with musical frequency in the NUMS group. The frequency of the musical signal ranged between 0 and 20,000 HZ and mostly below 1000 (Figure 4b). In contrast, in the US group, cells are subjected to a constant stretch of 0.850% (49.73 μm) when 1 kV voltage was applied without musical signal input. Cells cultured on the musical dish without any stretch stimulation served as a control group.

After the stimulation for 4 days, cell morphology of the NUMS group and US group was similar with the control group. Cell arrays were nondirectional. Cell protrusions became shorter, and the cytoplasm and nucleus gradually enlarged (Figure S2). Quantitative analysis showed that there was no significant difference in cell area and aspect ratio after stimulation in the NUMS and US groups, compared with the control group (Figure 4c,d). The aspect ratio of cells in the three groups decreased gradually with the increase of time. These results showed that the NUMS and US of music dish had no significant effect on the morphology of cells.

As external environment and cell cytoskeleton arrangement act in concert to regulate cell fate,^23^, ^24^ we analyzed changes in cytoskeleton after stimulation for 6 h. Compared with the control group, cells in the NUMS and US groups showed more obvious filamentous stress fibers, distributing through the whole cells and arranging along the long axis of the cells (Figure 4e). Fluorescence intensity of stress fibers staining was significantly higher in the NUMS group than the US and control groups (Figure 4f). The results suggested that NUMS was beneficial to the formation of stress fibers.

### 2.7 The effect of stretch stimulation on transcriptomic profiles

**FIGURE 5:**
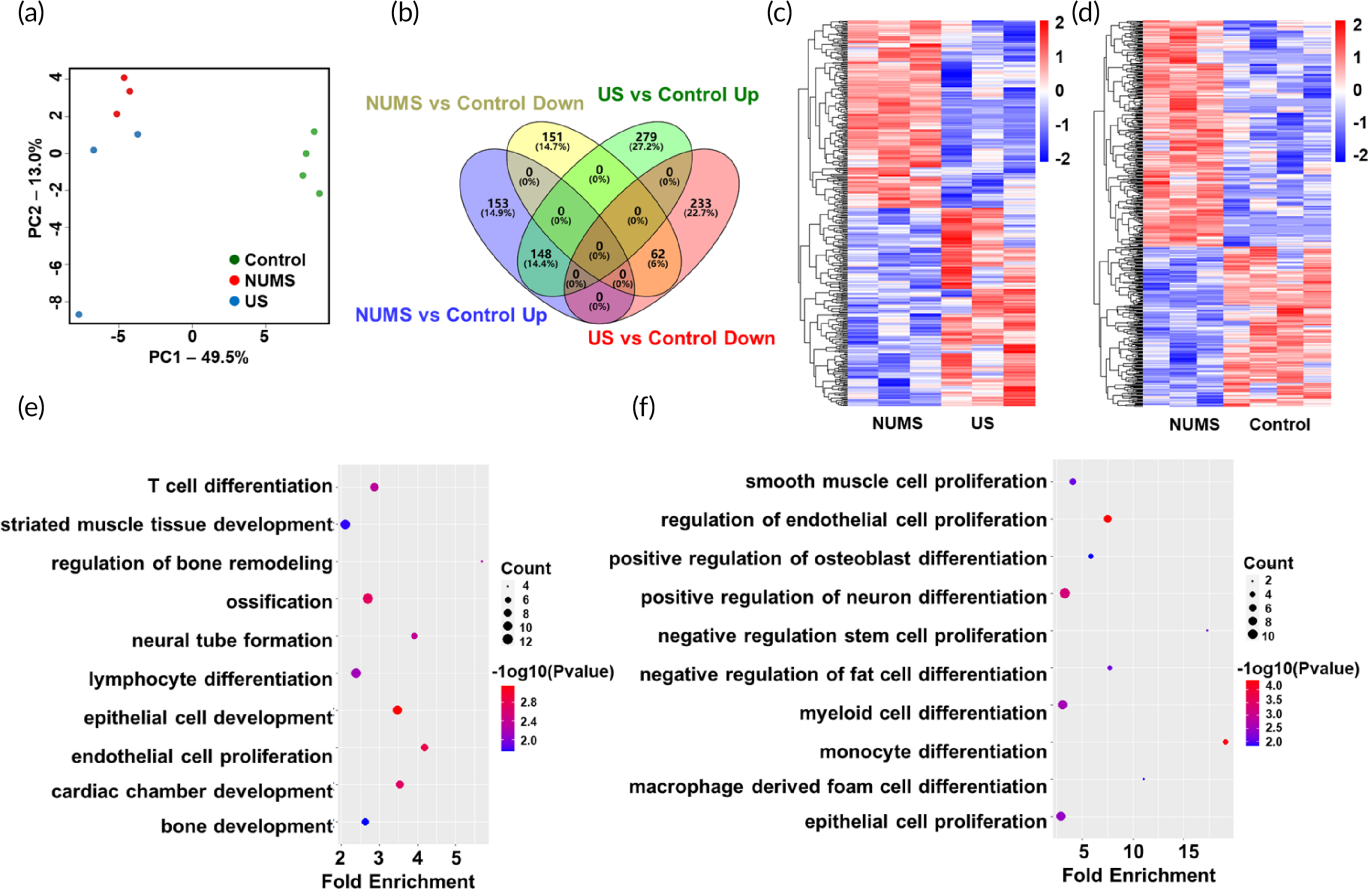
The effect of stretch stimulation on transcriptomic profiles. (a) Principal component analysis. (b) Venn diagram of differentially expressed genes (DEGs). (c) Heatmap of differentially expressed genes between the nonuniform microstretching (NUMS) and uniform stretch (US) groups. (d) Heatmap of differentially expressed genes between the NUMS and control groups. (e,f) Up‐regulated gene ontology (GO) terms between the NUMS and control groups (e), as well as the NUMS and US groups (f)

To investigate gene expression changes, a whole transcriptome sequencing was performed on cells in the NUMS, US, and control groups after stimulation for 9 days. Principal component analysis (PCA) revealed a close clustering of the NUMS and US groups, as well as a clear separation between the treatment groups and control group (Figure 5a). Pairwise comparisons were performed to reveal differentially expressed genes (DEGs) between each group. Compared with the control group, 722 and 514 DEGs were identified in the NUMS and US groups, respectively. Among these genes, 148 and 62 genes were up‐ and down‐regulated in both groups compared with the control group, respectively, while no genes showed opposite regulatory trends in these two groups (Figure 5b). Moreover, 197 genes were up‐regulated and 207 genes were down‐regulated in the NUMS group when compared to the US group (Figure S3A, S3B). Heatmaps showed the significant differences in gene expression between the NUMS and US groups, the NUMS and control groups, as well as the US and control groups (Figure 5c,d and Figure S3C).

To gain further functional insights, the gene ontology (GO) enrichment analysis was performed. The top 20 significantly enriched items of GO terms between the NUMS and control group included triglyceride catabolic process, neutral lipid catabolic process, acylglycerol catabolic process, pyruvate metabolic process, ADP metabolic process, glycolytic process (Figure S4A). The GO terms between the NUMS and US group were significantly enriched in chromatin‐mediated maintenance of transcription, cellular response to vitamin D, necroptotic process, plasma lipoprotein particle assembly, protein–lipid complex assembly, regulation of platelet‐derived growth factor receptor signaling pathway (Figure S4B).

We then focused on the GO terms related to cell fate regulation. In the US group, skeletal muscle tissue regeneration and myeloid cell development were significantly up‐regulated compared with the control group, while myeloid cell differentiation was up‐regulated compared with the NUMS group (Figure S5A‐B). In the NUMS group, up‐regulated GO terms included regulation of bone remodeling, ossification and bone development compared with the control group (Figure 5e), and positive regulation of cell differentiation, such as osteoblast and neuron, was also up‐regulated compared with the US group (Figure 5f). These findings indicated that different patterns of mechanical stimulation by our device may have distinct effects on stem cell differentiation potential.

### 2.8 The effect of stretch stimulation on cell differentiation

**FIGURE 6:**
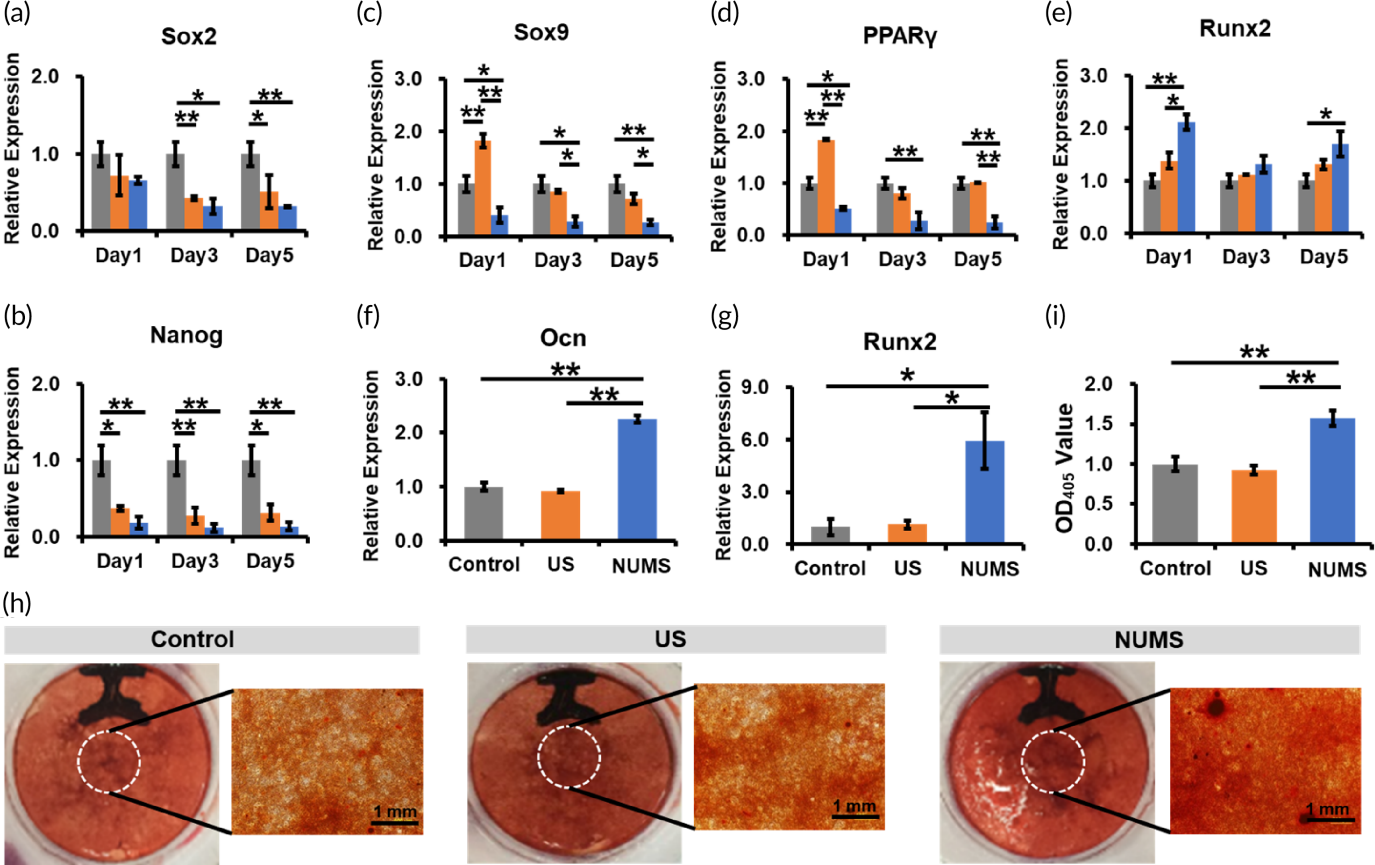
The effect of stretch stimulation on cell differentiation. (a–e) Q‐PCR analysis of the expression level of genes related to stemness (a,b), chondrogenesis (c), adipogenesis (d) and osteogenesis (e) after stimulation (*N* = 3, *n* = 3). (f,g) Q‐PCR analysis of the expression level of genes related to osteogenesis after osteogenic induction (*N* = 3, *n* = 3). (h,i) Alizarin red staining (h) and quantitative analysis (i) after osteogenic induction (*N* = 3, *n* = 3)

To further investigate cell fate determination, genes related to cell fate were tested after stimulation for 9 days. The results showed that the expression of stemness‐related genes *Sox2* and *Nanog* decreased significantly in the NUMS and US groups than that in the control group (Figure 6a,b). Compared with the control group, the expression of chondrogenic differentiation‐related gene *Sox9* and adipogenic differentiation‐related gene *PPARγ* increased significantly in the US group, while the expression of osteogenic differentiation‐related gene *Runx2* remained unchanged (Figure 6c,e). However, the expressions of *Sox9* and *PPARγ* were significantly down‐regulated and *Runx2* was significantly up‐regulated in the NUMS group, compared with the US and control group. These results showed that cells would differentiate into mature cells after stretch stimulation, and the NUMS promoted osteogenic differentiation of cells, the US promoted adipogenic and chondrogenic differentiation at the early stage.

Next, we cultured cells in osteogenic induction medium to explore whether the NUMS can enhance the osteogenic effect under induction condition. After inducing for 21 days, the expression level of osteogenic differentiation‐related genes *Runx2* and *Ocn* was detected. The results showed that the expression of *Runx2* and *Ocn* increased significantly after the NUMS stimulation compared with the US and control groups but did not change significantly after the US stimulation (Figure 6f,g). The results of alizarin red staining showed typical mineralized nodules were formed in the NUMS group, while atypical mineralized nodules were formed in both the US and control groups (Figure 6h). Quantitative results showed that the NUMS group had significantly higher alizarin red staining compared to the US group and control groups (Figure 6I). These results further demonstrated that the NUMS can promote osteogenic differentiation of cells.

## 3 CONCLUSION

Our findings highlight a newly developed device, “musical dish,” which allows arbitrary strain waveforms to be applied to cells by converting music signal into stretching force (Figures 1a,b and 3a). A bionic NUMS was subjected to cells through the musical dish (Figure 4a,b). The NUMS involved both increased cytoskeletal tension via stress fiber formation and enhanced *Runx2* signaling that contributes to osteogenic differentiation of cells. First, the initial in vitro culture studies examined cell morphology and cell cytoskeleton, as both of them have been correlated with cell fate. Although there were no significant changes in cell morphology among the NUMS, US, and control groups, it was found that more stress fibers were formed after a short time of NUMS stimulation (Figure 4c–f). Whole transcriptome sequencing was used to gain insight into gene expression profiles of cells treated by three different stretch stimulation. GO enrichment analysis illustrated that the NUMS group had more significant biological effects on the bone development associated pathways than the US and control group (Figure 5e,f). To further support this observation, expression changes in cell fate‐related genes was detected. In the NUMS group, the expression of osteogenesis‐related gene *Runx2* was up‐regulated, and stemness, adipogenesis, and chondrogenesis‐related genes were down‐regulated (Figure 6a–e). Furthermore, after induction by osteogenic medium, the NUMS significantly promoted osteogenic differentiation of cells to form typical mineralized nodules, while the US group showed the same inconspicuous staining results as the control group (Figure 6h). The results of osteogenic differentiation promotion effect of the NUMS were consistent with the sequencing analysis results.

## 4 DISCUSSION

The reason for the lack of significant difference in cell morphology between the groups could be due to the small stretch amplitude in the NUMS and US groups. The effect of stretch on morphological alteration depends on strain amplitude, frequency, and duration.^25^ Thresholds of these parameters that can affect cell morphology vary in different studies. For example, cells exposed to a stretch treatment with an amplitude of 10% and a frequency of 1 Hz showed similar shape index with the control group without any stretching.^26^ Under 5% stretching, the aspect ratio of the cells showed only a small change compared with the control group without stretch.^27^ Another study showed that stretch with 5% amplitude and 1 Hz frequency did not change the morphology of cells.^28^ It has been reported that even if cells were stimulated by 1% and 2.75 Hz cyclic stretch, they showed similar area and shape index with the control group without stretch.^29^ Here, the stretch amplitude of the NUMS and US is 0.85%. Thus, we believe that the aspect ratio will not change significantly under the stretch induced by such a low amplitude.

Although cell morphology is closely related to cell fate, changes in cell morphology are not a necessary for cell fate regulation. For example, cells under 10% and 1 Hz stretch showed similar morphology with the untreated group, but the stretch induced tenogenic differentiation of the cells.^26^ Another study found that when cells encapsulated in hydrogels with different stiffness showed similar morphology, cells fate can also be regulated by the stiffness.^30^ Previous studies have found that, in addition to maintaining cell morphology, cytoskeleton participated in the transmission of mechanical signals, which changed the activation, synthesis, secretion, and other functions of corresponding effector molecules, thus affecting cell proliferation, differentiation, movement, and other activities.^31^, ^32^ In addition, the cytoskeleton links to Lamin A through the LINC complex to anchor the chromatin. Therefore, cytoskeletal tension has the potential to directly alter gene expression.^33^

The GO analysis results also showed that neural differentiation was enriched in the NUMS group compared with the US group (Figure 5f). There is accumulating evidence suggesting “neuro” related processes can shed light on “osteo” related processes. For examples, *Runx2* is functionally connected to many genes that are important for brain and language development, but also to bone formation.^34^ *Runx2* directly interacts with *Satb*2, a gene that regulates stereotypic projections in the cortex.^35^ This gene also plays a key role in osteoblast differentiation.^36^ Moreover, *Runx2* interacts with *Dyrk1a*, a gene that not only controls neural precursor activity and differentiation but also involves in bone homeostasis as an inhibitor of osteoclastogenesis.^37^, ^38^ Other neurogenetic factors such as *Cbl*, *Foxp2*, *Hes1*, and *Akt1* were also reported to interact with *Runx2*.^39^, ^40^, ^41^, ^42^ Based on the crosstalk between the osteogenic and neurogenic stem cells, we believe that the NUMS may have great potential to promote bone differentiation of cells.

For clinical application, three strategies may contribute to the development of potential therapeutic ways for bone regeneration. First, the NUMS could be used in the DO surgery to replace or assist the current large‐scale and slow stretching method for bone regeneration due to its excellent performance in promoting osteogenic differentiation. The second is the “musical dish,” which could be further developed into a music derived rehabilitation technology to promote bone healing. Finally, the musical dish can serve as a cell expansion bioreactor to produce cells with high level of osteogenic differentiation to repair bone repair by transplantation. Thus, both the new stretch model and the newly developed device for stretching may have a great potential for clinical application.

## 5 MATERIALS AND METHODS

### 5.1 Fabrication of the musical dish

A poly methyl methacrylate (PMMA) ring was used as the rigid frame of the dish. The outer and inner radiuses of the PMMA ring were 40 mm and 30 mm, respectively. An acrylic‐based copolymer elastomer film (VHB 4910, 3 M, USA) with an initial thickness of 1 mm and prestretched radially by 200% was used as the DE membrane. The resulting thickness was about 0.1 mm. The soft electrode was a thin layer of SWCNTs (Aladdin, China). The fabricating process of the SWCNT electrode was presented by Shian et al.,^43^ including (1) preparing stable colloidal suspensions of SWCNTs; (2) forming percolation mat by filtration; and (3) transferring the percolation mat onto the prestretched VHB membrane. After the SWCNT electrode was transferred to VHB membrane, the VHB membrane was sandwiched by two PMMA rings to form a DEA stretcher. The fabricated DEA stretcher was fixed to a hollowed‐out dish (Corning, USA) to form the musical dish. Thin strips of tinfoil were used as conductive tape for the cables of the high‐voltage power supply. Graphite carbon paste was used to guarantee the tight contact between the SWCNT electrode and the tinfoil. At last, to protect the SWCNT electrode, a thin layer (about 50 μm) of polydimethylsiloxane (PDMS) (Sylgard 184, Dow Corning) was spin coated at 4000 rpm. To reduce the stiffening effect of the PDMS to the VHB membrane, a 20:1 monomer‐curing agent ratio was adopted. The temperature and time for curing were set to be 40°C and 12 h.

### 5.2 Strain characterization of the musical dish

The actuating strains of the stretcher under different applied voltages were measured. Potable natural mineral water (NONGFU SPRING) was used as the ground electrode. The voltages (3, 4, and 5 kV) were generated by a high‐voltage power supply (610E, TREK, USA). A DSLR camera (70d, Cannon, Japan) was used to capture the profiles of the SWCNT electrode during actuation. Considering the viscoelasticity of VHB membrane, each picture was shot 1 min after the onset of the voltage. And before a new voltage was applied, there was 1 min for the stretcher to recover to its initial state. The radial strain was then calculated as:

(1) $$\varepsilon_{r}=\frac{∆r}{r_{0}}$$

where $r_{0}$ is the initial radius of the electrode and ∆r is the difference of the radiuses before and after the voltage was applied. The radius of the electrode during actuating was measured as half the distance between point A and point B, as shown in Figure 1d. The procedure was repeated nine times and there was 10 min for relaxation each time after the voltage is off.

To assess the actuating stability, two square‐wave driving signals at 0.15 Hz and 1 Hz with a 50% duty cycle were generated respectively by a function signal generator (AFG1022; Tektronix, USA) which was connected to the high‐voltage power supply (610E, TREK). The stretcher was then cycled between 0 and 5 kV for 4 h. At specific time points (0, 10, 30, 120, and 240 min), a 1 min period of video was shot to record the dynamic response of the stretcher. The radial strain, calculated by Equation (1), was taken as the average value in that minute.

### 5.3 Model analysis of the homogeneity and equiaxiality of the musical dish

The active part (electrode covered area, including the central circular zone and the electrode lead zone) shrinked in thickness under the Maxwell stress:

(2) $$\;\sigma_{\max}=\varepsilon_{0}p_{r}E^{2}=\varepsilon_{0}p_{r}{\left(V/t\right)}^{2}$$

where $\varepsilon_{0}$ is the permittivity of vacuum, $p_{r}$ is the dielectric constant of the elastomer, *E* is the electric field, *V* is the applied voltage, and *t* is the thickness of the DE membrane.

Commercially available finite element analysis (FEA) package ABAQUS was used to obtain the strain distribution of the active part of the VHB membrane. The geometric parameters of the model were set to be the values before prestretch. Yeoh model was used to describe VHB's hyperelasticity and incompressibility. The Yeoh form of strain energy potential was described as:

(3) $$\;W=C_{10}\left(I_{1}-3\right)+C_{20}{\left(I_{1}-3\right)}^{2}+C_{30}{\left(I_{1}-3\right)}^{3}$$

where $I_{1}$ is the first invariant of the left Cauchy‐Green deformation tensor and $C_{10}$, $C_{20}$, $C_{30}$ were three material parameters. The material parameters of VHB 4910 were listed in Table 1.^44^

**TABLE 1:** Material parameters of VHB 4910

Parameters	Unit	Value
$C_{10}$	MPa	0.0693
$C_{20}$	MPa	−8.88 × 10^−4^
$C_{30}$	MPa	16.7 × 10^−6^
$\varepsilon_{0}$	As/Vm	8.85 × 10^−12^
$p_{r}$	‐	4.7

The modeling process was composed of two steps. In the first step, a displacement was applied to stretch the membrane by 200% radially. In the second step, a pressure was imposed to squeeze the active part in thickness. The amplitude of the pressure (with the applied voltage of 5 kV) was calculated by Equation (2). This approximation was well suited for planar actuators.^44^, ^45^, ^46^ The electric field was calculated based on the initial thickness (the thickness after prestretch) of the membrane instead of the true thickness under actuation. This led to a lower applied electric field intensity compared with that in the equilibrium state. However, the calculated Maxwell stress under 5 kV could be treated as the value under a lower applied voltage. Considering that only the homogeneity of the strain distribution was desired, it was not necessary to figure out what the exact value of the applied voltage was. The model consisted of 5164 linear brick elements with hybrid formulation (C3D8H).

### 5.4 Assembling musical dishes in a culture system

The developed culture system includes a musical dish, a CO_2_ incubator (Heal Force, H90, China), an audio power amplifier (XianKe, SU‐120(MV‐70), China), a voltage power supply (Trek, 610E‐K‐CE) (Figure 3a). Musical signal input with playing the song “Vitas‐Opera‐2” (Supplementary materials 1).

### 5.5 Linear strain of musical dish

Linear strain was measured by the same method as “radial strain” to describe the stretchability of the musical dish. Cell culture medium was used as the ground electrode. A series of constant voltages of 0.5, 1, 1.5, 2, 2.5, 3, 3.5, 4, 4.5, and 5 kV were applied. The linear strain (w%) was calculated by a formula:

(4) $$w\%=\varepsilon_{r}\times 100\%$$

where *ε*_*r*_ is the radial strain.

### 5.6 Cell culture and proliferation

The mouse cell line C3H10T1/2 was obtained from Cell Bank of the Chinese Academy of Science. The cells were cultured in incubator at 37°C with 5% CO_2_. Proliferation medium comprised of low glucose Dulbecco's modified Eagle's medium (DMEM, GIBCO, USA) supplemented with 10% fetal bovine serum (FBS, GIBCO), and 1% penicillin–streptomycin (PS, GIBCO). The medium was replaced every 2 days. When the cells were 80%–90% confluent, they were passaged by 0.05% trypsin (GIBCO) at 37°C for 3 min. Cells were observed and photographed by inverted microscope (Olympus, Japan).

Cell proliferation was measured by the Cell Counting Kit‐8 (CCK‐8, DOJINDO, Japan). Briefly, cells were seeded on dishes at a density of 5000 cells/cm^2^. After culturing for 1, 3, and 5 days, cell medium was replaced by fresh medium containing 10% CCK‐8 reagent. After incubating for 2 h in CO_2_ incubator, the absorbance was tested by microplate reader (Spectra‐max190, USA) at 450 nm.

### 5.7 Cell morphology and cell cytoskeleton

Cell area and aspect ratio were used to evaluate cell morphological change. From Day 0 to Day 4, cell morphology was observed and photographed by inverted microscope (Olympus) in every day. The cell area and aspect ratio were analyzed by image J.

The cytoskeleton and the nuclei of cells were stained by phalloidin with FITC or TRITC labeling (Cytoskeleton, USA) and DAPI (Biyuntian, China), respectively. Briefly, proliferation medium was discarded. Cells were washed by phosphate‐buffered saline (PBS) for three times and fixed with 4% paraformaldehyde for 10 min. The fixed cells were rinsed three times by PBS. 2% phalloidin dye was added and incubated for 1 . After washing for three times by PBS, 0.1% DAPI dye was added and incubated for 10 min. After washing for three times by PBS, cell morphology was visualized using inverted fluorescence microscope (Olympus).

### 5.8 RNA sequencing

For RNA sequencing, total RNA was extracted by TRIzoL rapid extraction method. Briefly, after the medium was discarded, 1 ml Trizol (Takara, Japan) was added into dishes. Cells were transferred to 1.5 ml EP tubes with Trizol. A 0.2 ml of chloroform (Sinopharm, China) was added and mixed for 15 min, followed by centrifugation at 12,000 g, 4°C for 10 min. Take the top clear liquid to a new tube. Isopropyl alcohol (Sinopharm) was added at a ratio of 1:1 and mixed for 10 min, followed by centrifugation at 12,000 g, 4 °C for 10 min. Discard the supernatant and added 1 ml of 75% ethylalcohol (Sinopharm), followed by centrifugation at 12,000 g, 4°C for 10 min. Discard the supernatant and dry for 5–10 min. RNA was dissolved by 20 μl of distilled water.

RNA sequencing was performed as described before.^47^ The extracted RNA was reverse transcribed by SuperScript II reverse transcriptase (Invitrogen), double‐strand cDNA was conducted using NEBNext mRNA second strand synthesis kit (NEB), double‐strand DNA was cleaned with AMPure XP beads (Beckman Coulter), sequencing library was constructed with Nextera XT kit (Illumina) and sequenced on Illumina X‐Ten platform. Sequence reads were mapped to reference genome mm10 using Bowtie2 using default parameters and per gene counts were calculated using HTSeq. R statistical programming language was utilized to analyze all statistical data. DESeq2 was used to identify differentially expressed genes (DEGs). In our evaluation, a gene was considered to be expressed in a sample if its count value was equal or greater than 1 in the sample. DEGs were illustrated as fold‐change ≥2 and *p* value ≤ 0.05. GO analysis was performed using DAVID (http://david.ncifcrf.gov).

### 5.9 Quantitative RT‐PCR analysis

The total RNA was extracted by TRIzoL rapid extraction method as above. RNA was reverse transcribed using 5 × RT Master Mix (TOYOBO, Japan) and 2 μg RNA template. The amplification procedure was used as follow: 37°C for 15 min; 50°C for 5 min and 98°C for 5 min in PCR Amplifier (Biometra, Germany). RT‐PCR was performed using 25 ng of cDNA samples using 5 mmol/L of each primer (Table 2) and 5 μl of Brilliant SYBR Green QPCR Master Mix (Takara, Japan) in a Light Cycler apparatus (Roche, Switzerland). The amplification procedure was used: 95°C for 5 min; 40 cycles at 95°C for 5 s; 60 °C for 30 s. All values were normalized to the GAPDH housekeeping gene and relative expressed as fold change using the formula 2^−△△Ct^. The primers for RT‐PCR are listed in Table 2.

**TABLE 2:** primers for RT‐PCR

Gene	5′–3'	Primer	Size (bp)
Mouse SOX2	Forward	CTGGACTGCGAACTGGAGAAG	67
	Reverse	TTTGCACCCCTCCCAATTC	
Mouse Nanog	Forward	TTGAAGACTAGCAATGGTCTGAT	125
	Reverse	TGGCTGCCCCACATGGAAAGG	
Mouse Runx2	Forward	TGACATCCCCATCCATCCAC	120
	Reverse	AGAAGTCAGAGGTGGCAGTG	
Mouse Ocn	Forward	TGCTTGTGACGAGCTATCAG	149
	Reverse	GAGGACAGGGAGGATCAAGT	
Mouse Sox9	Forward	GAGGCCACGGAACAGACTCA	51
	Reverse	CAGCGCCTTGAAGATAGCATT	
Mouse PPARγ	Forward	CAGTTGATTTCTCCAGCATTTCT	124
	Reverse	ACTTTGATCGCACTTTGGTATTC	
Mouse Gapdh	Forward	GCAAGTTCAACGGCACAG	141
	Reverse	CDCCAGTAGACTCCACGAC	

### 5.10 Osteogenic differentiation

To verifying the osteogenic differentiation effect of stimulation for cells, differentiation medium comprised of high DMEM (GIBCO) supplemented with 10% FBS, 1% PS, 10 mM β‐sodium glycerophosphate (Sigma, Germany), 10^−5^ mM dexamethasone (Sigma) and 50 μg/ml of ascorbic acid (Sigma) was used. Alizarin red (ARS, Sangon, China) was used to characterize osteogenic differentiation of cells. After culturing for 21 days, medium was discarded. Cells were washed by PBS for three times and fixed with 4% paraformaldehyde (SINOPHARM, China) for 30 min. The fixed cells were rinsed three times by distilled water. 2% ARS was added to stain for 30 min. Then cells were washed by distilled water for five times to stop reaction. The result of staining was visualized using inverted microscope (Olympus). For quantitative analysis, 0.5 M HCl (Sinopharm) containing 5% SDS (Sigma) was added to elute the dye. After 30 min, the absorbance of supernatant was tested by microplate reader (Spectra‐max190) at 405 nm.

### 5.11 Statistical analysis

In this experiment, all data were expressed as mean standard deviation, and their statistical significance was evaluated by *t*‐test. The *t*‐test was carried out with the function of T.TEST in Microsoft Excel. Single asterisk (*) expresses significant differences between groups (0.01 < *p* < 0.05), double asterisk (**) expresses significant differences between groups (*p* < 0.01).

## AUTHOR CONTRIBUTIONS

**Qiulin He:** Data curation (equal); methodology (equal); visualization (equal); writing – original draft (equal). **Junxin Lin:** Data curation (equal); methodology (equal); visualization (equal). **Fanghao Zhou:** Data curation (equal); methodology (equal); resources (equal); visualization (equal). **Dandan Cai:** Data curation (equal); methodology (equal); visualization (equal). **Yiyang Yan:** Data curation (equal); methodology (equal); visualization (equal). **Yejie Shan:** Data curation (equal); methodology (equal); resources (equal); visualization (equal). **Shufang Zhang:** Writing – review and editing (equal). **Tiefeng Li:** Conceptualization (equal); resources (equal). **Xudong Yao:** Writing – review and editing (equal). **Hongwei Ouyang:** Conceptualization (lead); writing – review and editing (lead).

6

### PEER REVIEW

The peer review history for this article is available at https://publons.com/publon/10.1002/btm2.10291.

## Supporting information


**Appendix** **S1.** Supporting Information.
