# Supplementary material for: “Musical dish” efficiently induces osteogenic differentiation of mesenchymal stem cells through music derived microstretch with variable frequency
Source: Bioeng Transl Med. 2022 Jan 25;7(2):e10291. doi: 10.1002/btm2.10291 (PMC9115692; doi:10.1002/btm2.10291)
Supplement: Supplementary file 1 — Appendix S1. Supporting Information. [file BTM2-7-e10291-s001.docx]

**Supplementary materials**


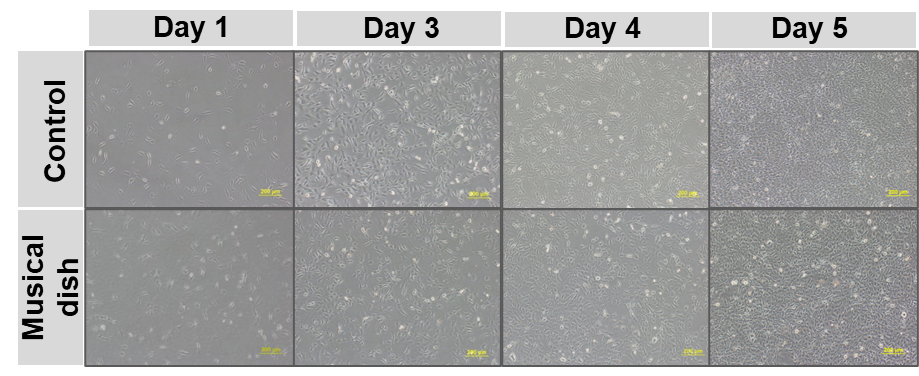


Figure S1. Pictures of cells in the musical dish and petri dish groups from day1 to day5 under inverted microscope.


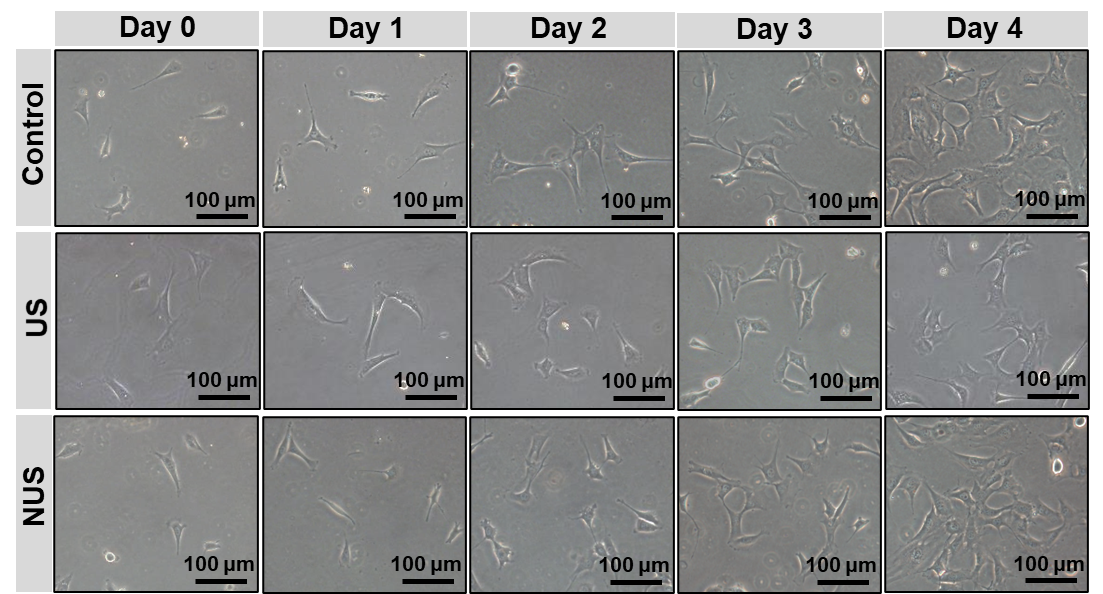


Figure S2. Pictures of cells in the NUMS, US and control group from day1 to day4 under inverted microscope.


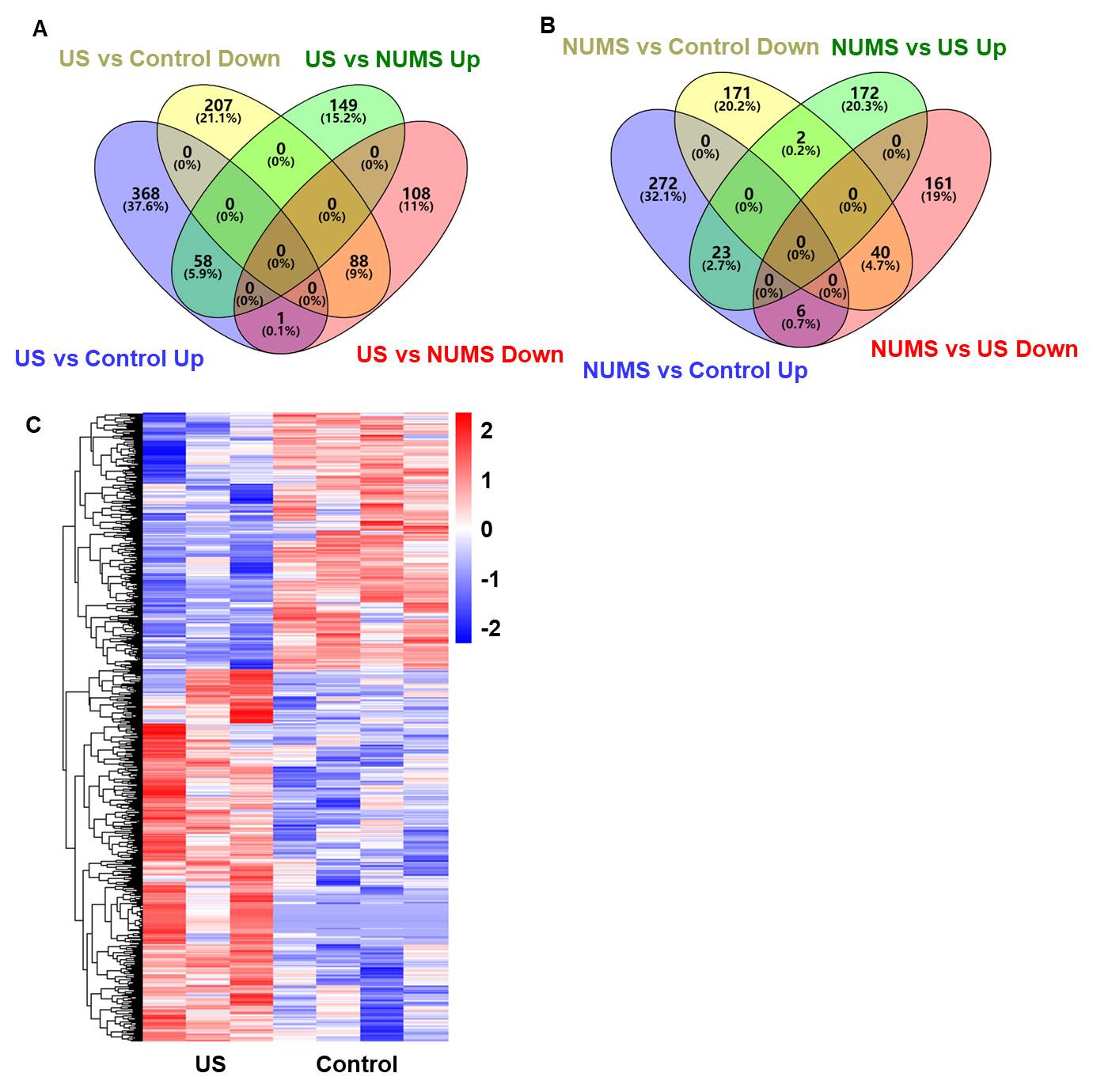


Figure S3. (A-B) Venn diagram of DEGs. (C) Heatmap of differentially expressed genes between the US and control.


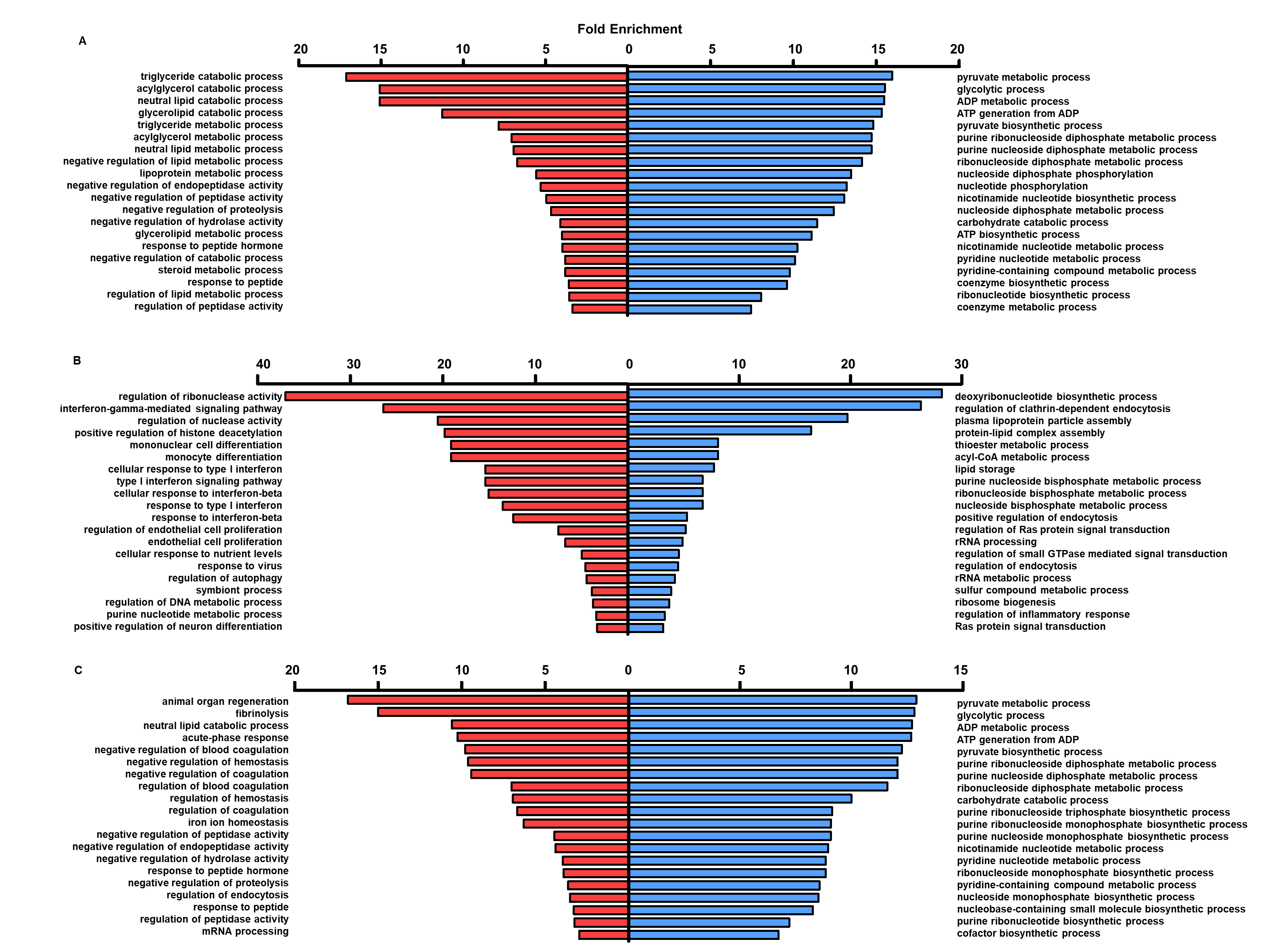


Figure S4. GO terms of DEGs between the NUMS and Control groups (A), the NUMS and US groups (B), as well as the US and Control groups (C). Red: up-regulated, Bule: down-regulated.


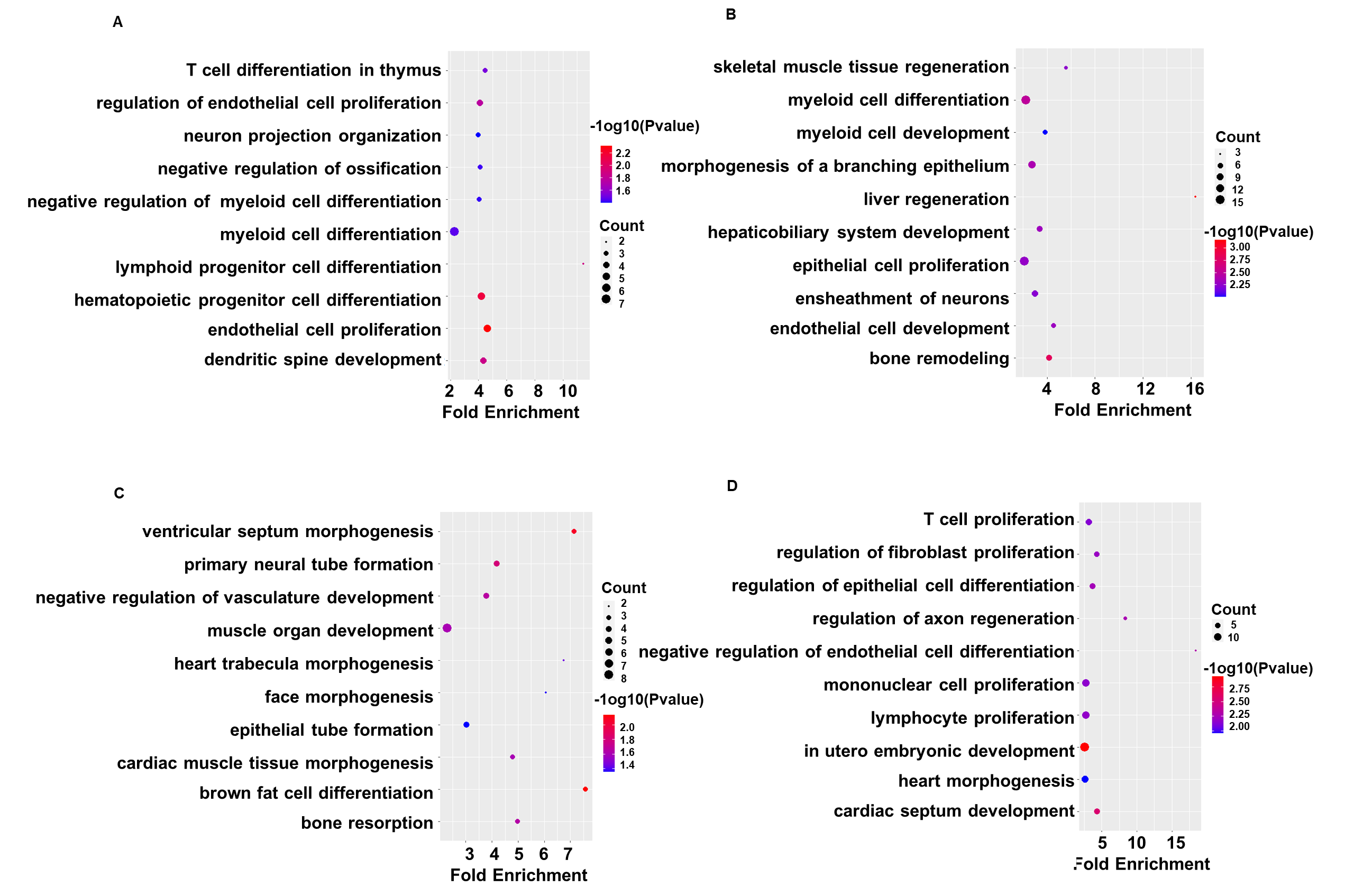


Figure S5. GO terms of NUMS vs US down-regulated (A), US vs Control up-regulated (B), NUMS vs Control down-regulated (C), US vs Control down-regulated (D).
